# Periodontal ligament stem cell-based bioactive constructs for bone tissue engineering

**DOI:** 10.3389/fbioe.2022.1071472

**Published:** 2022-12-02

**Authors:** Zeqing Zhao, Jin Liu, Michael D. Weir, Abraham Schneider, Tao Ma, Thomas W. Oates, Hockin H. K. Xu, Ke Zhang, Yuxing Bai

**Affiliations:** ^1^ Department of Orthodontics, School of Stomatology, Capital Medical University, Beijing, China; ^2^ Key Laboratory of Shaanxi Province for Craniofacial Precision Medicine Research, College of Stomatology, Xi’an Jiaotong University, Xi’an, China; ^3^ Biomaterials and Tissue Engineering Division, Department of Advanced Oral Sciences and Therapeutics, University of Maryland Dental School, Baltimore, MD, United States; ^4^ Department of Oncology and Diagnostic Sciences, University of Maryland School of Dentistry, Baltimore, MD, United States; ^5^ Marlene and Stewart Greenebaum Cancer Center, University of Maryland School of Medicine, Baltimore, MD, United States; ^6^ Center for Stem Cell Biology and Regenerative Medicine, University of Maryland School of Medicine, Baltimore, MD, United States

**Keywords:** periodontal ligament stem cells, scaffold, bone regeneration, periodontal tissue regeneration, tissue engineering

## Abstract

**Objectives:** Stem cell-based tissue engineering approaches are promising for bone repair and regeneration. Periodontal ligament stem cells (PDLSCs) are a promising cell source for tissue engineering, especially for maxillofacial bone and periodontal regeneration. Many studies have shown potent results *via* PDLSCs in bone regeneration. In this review, we describe recent cutting-edge researches on PDLSC-based bone regeneration and periodontal tissue regeneration.

**Data and sources:** An extensive search of the literature for papers related to PDLSCs-based bioactive constructs for bone tissue engineering was made on the databases of PubMed, Medline and Google Scholar. The papers were selected by three independent calibrated reviewers.

**Results:** Multiple types of materials and scaffolds have been combined with PDLSCs, involving xeno genic bone graft, calcium phosphate materials and polymers. These PDLSC-based constructs exhibit the potential for bone and periodontal tissue regeneration. In addition, various osteo inductive agents and strategies have been applied with PDLSCs, including drugs, biologics, gene therapy, physical stimulation, scaffold modification, cell sheets and co-culture.

**Conclusoin:** This review article demonstrates the great potential of PDLSCs-based bioactive constructs as a promising approach for bone and periodontal tissue regeneration.

## Introduction

Bone defects due to congenital malformations, skeletal diseases, trauma, inflammation and tumor resections pose a critical challenge. Auto grafts are deemed as the gold-standard for bone regeneration; however, their application is impeded by harvest limitations and donor-site morbidity. To meet this need, stem cell-based tissue engineering has emerged as a promising option to regenerate bone defect. Research of stem cells has been a fascinating area of interest. Because of the capability of self-renewal and differentiation into multiple cell lineages, stem cells were regarded as powerful roles in regeneration of lost tissues (Avinash et al., 2017). Mesenchymal stem cells (MSCs) have been identified by capabilities of forming adherent fibroblast-like colonies and differentiating into multiple lineages including osteogenesis, adipogenesis and chondrogenesis (Queiroz et al., 2021). MSCs possess immunomodulatory ability, migratory activity and paracrine function on other type of cells (Queiroz et al., 2021).

When isolated from diverse tissues, different types of MSCs demonstrate source-dependent peculiarities. In recent years, many dentally-derived MSCs were found suitable for tissue engineering applications because of their accessibility and multilineage differentiation capacity (Lei et al., 2014). As dentally-derived MSCs are cells originating from migrating neural crest cells, they play a strategic role in dental and craniofacial tissue regeneration (Trubiani et al., 2019).

Periodontal ligament stem cells (PDLSCs) are isolated from periodontal ligaments (PDL). PDLSCs can be harvested from the extracted wisdom teeth, extracted supernumerary teeth and the teeth extracted for orthodontic treatment (Zhao et al., 2021). PDLSCs are a relative easily accessible and low-cost source of stem cells, without extra invasive procedures (like bone marrow aspiration) (Zhao et al., 2021). PDLSCs can differentiate into osteoblasts, chondrocytes, cementoblasts and adipocytes *in vitro* and regenerate PDL-like tissues *in vivo* (Qiu et al., 2020). It has been found that, as a kind of MSCs, PDLSCs participate in bone repair *via* three ways: osteodifferentiation, release of cytokines and extracellular vesicles, and immunomodulatory function (Zha et al., 2022). It has been well known that the effect of tissue regeneration relies on the oxygen and nutrient support supplied by the local vasculature (Yeasmin et al., 2014). Therefore, in the area of tissue engineering and regenerative therapy, angiogenic process has been in the spotlight these years. So far, many studies have demonstrated that PDLSCs have angiogenic properties, definitely, the capacity to support the construction of a functional vasculature (Yeasmin et al., 2014; Zhao et al., 2021; Iwasaki et al., 2021). These investigations highlight the vascular potential of PDLSCs. These advantages make PDLSCs a very attractive cell population for regenerative therapies.

Many pilot studies, pre-clinical studies as well as clinical trials have shown promising results regarding the effectiveness and the safety of PDLSCs in bone regeneration (Queiroz et al., 2021). In this review, we discuss current *in vitro* and *in vivo* advances in PDLSC-based bioactive constructs for bone tissue and periodontal tissue regeneration. Furthermore, the effects of osteoinductive strategies on PDLSC differentiation and regeneration capabilities were also discussed.

## Delivery of PDLSCs with various types of scaffolds

Currently, the most frequently utilized xenogenic bone grafts are of bovine origin, while grafts derived from porcine bone have shown potential, due to their architectural and compositional similarities to human bone (Bow et al., 2019). PDLSCs showed highly efficient cell proliferation, together with osteogenic differentiation when they were seeded on Bio-Oss scaffold (inorganic deproteinized bovine bone minerals) and Dual Block scaffold (collagenated porcine cortico-cancellous bone scaffold) (Yu et al., 2014; Diomede et al., 2016; Mazzoni et al., 2017). The seeding of PDLSCs can further improve the regenerative capacity of Bio-Oss scaffolds and Dual Block scaffolds (Yu et al., 2014; Diomede et al., 2016; Mazzoni et al., 2017). However, as organic components within the matrix have been removed, these grafts generally lack osteoinductivity (Bow et al., 2019). Therefore, for xenogeneic bone grafts, further investigation is needed to enhance their osteoinductive characteristics, and coupling with bioactive components could be an attractive approach (Musson et al., 2019).

**FIGURE 1:**
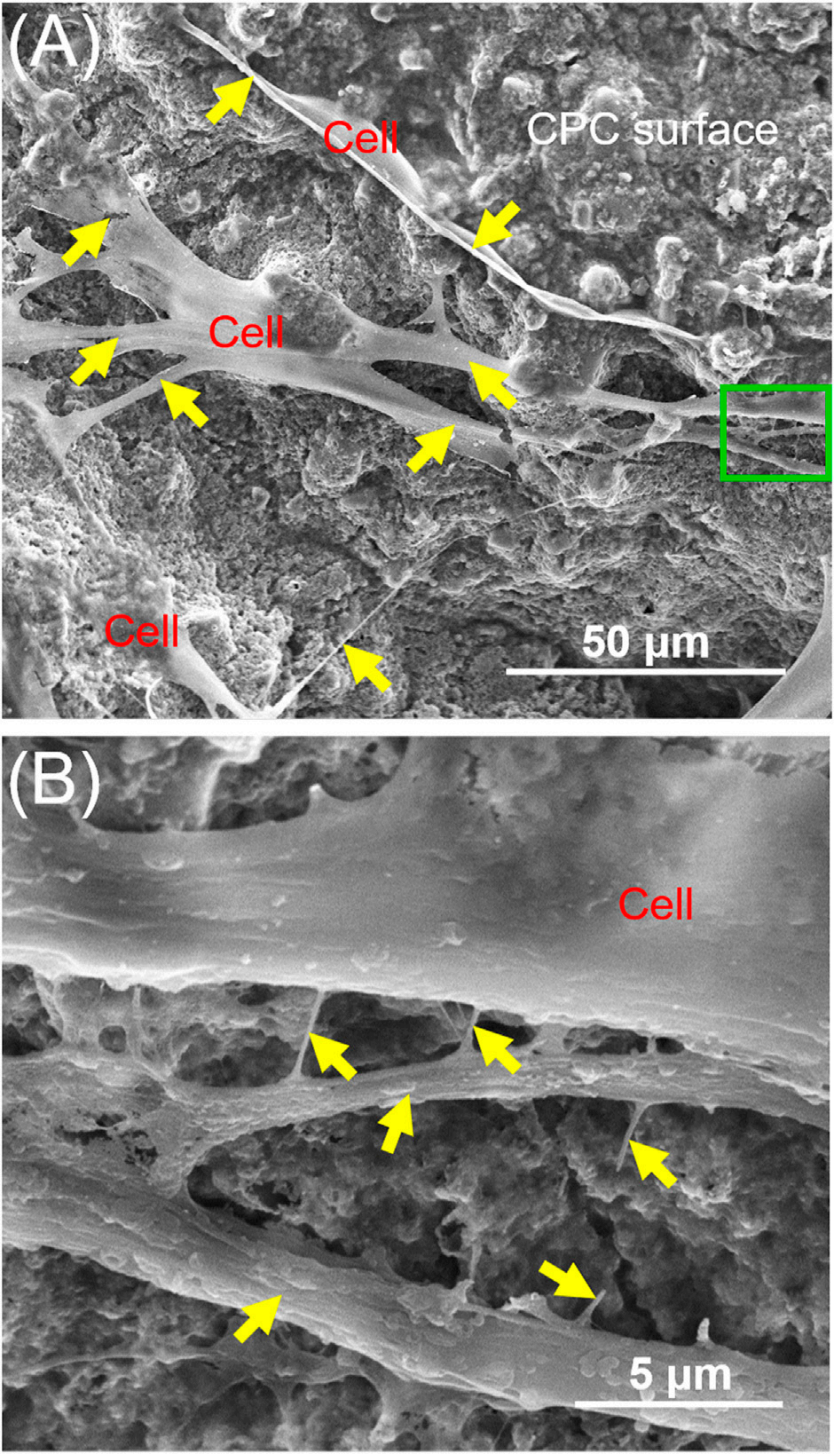
Typical SEM images of CPC scaffolds cultured with PDLSCs for 14 days. The image in **(B)** is a higher magnification of the green dotted frame in **(A)**. Yellow arrows indicate a healthy cell spreading morphology.

Hydroxyapatite (HA), tricalcium phosphate (TCP), biphasic calcium phosphate (BCP) and calcium phosphate cements (CPC) are also promising for bone regeneration applications. Calcium phosphate materials possess similar chemical characteristics to natural bone minerals (Chen et al., 2013; Mao et al., 2015; Sohn and Oh, 2019). They have been applied in bone tissue regeneration due to their excellent bioactivity and osteoconductivity (Chen et al., 2013; Mao et al., 2015; Sohn and Oh, 2019). PDLSCs adhered and stretched well on HA, TCP and BCP scaffolds, and kept their original osteogenic phenotypes (He et al., 2011; Mao et al., 2015; Su et al., 2015; Shi et al., 2018; Sohn and Oh, 2019). The combination of PDLSCs with HA, TCP and BCP significantly promoted effective bone regeneration (He et al., 2011; Mao et al., 2015; Su et al., 2015; Shi et al., 2018; Sohn and Oh, 2019). One CPC consisted of tetracalcium phosphate and dicalcium phosphate anhydrous (Zhou et al., 2012). The injectability and body-temperature reactions are important characteristics of CPC, which facilitates its ability to mold and fill the bone defect (Sohn and Oh, 2019). CPC can bond to bone tissues to form a functional interface (Chen et al., 2013). Zhao et al. (2019b) seeded PDLSCs on CPC scaffolds, demonstrating that CPC supported PDLSCs attachment and proliferation (Figure 1). Chitosan can be used as a viscous binder to rendered CPC fast-setting and increase the load-bearing capability (Wang et al., 2016). CPC scaffold containing chitosan is an effective delivery vehicle for drugs and proteins (Zhao et al., 2019b; Zhao et al., 2019c). Qin et al. (2018) and Qiu et al. (2021) revealed that CPC-chitosan scaffolds can sustainably release small molecular drug and growth factors. Metformin (a small molecular glucose lowering drug) and platelet lysate (HPL) carried by CPC-chitosan scaffold can be sustainably released for 15–21 days (Qin et al., 2018; Qiu et al., 2021). Metformin and HPL released by CPC increased the osteogenesis of PDLSCs seeded on CPC scaffolds (Qin et al., 2018; Zhao et al., 2019c).

**FIGURE 2:**
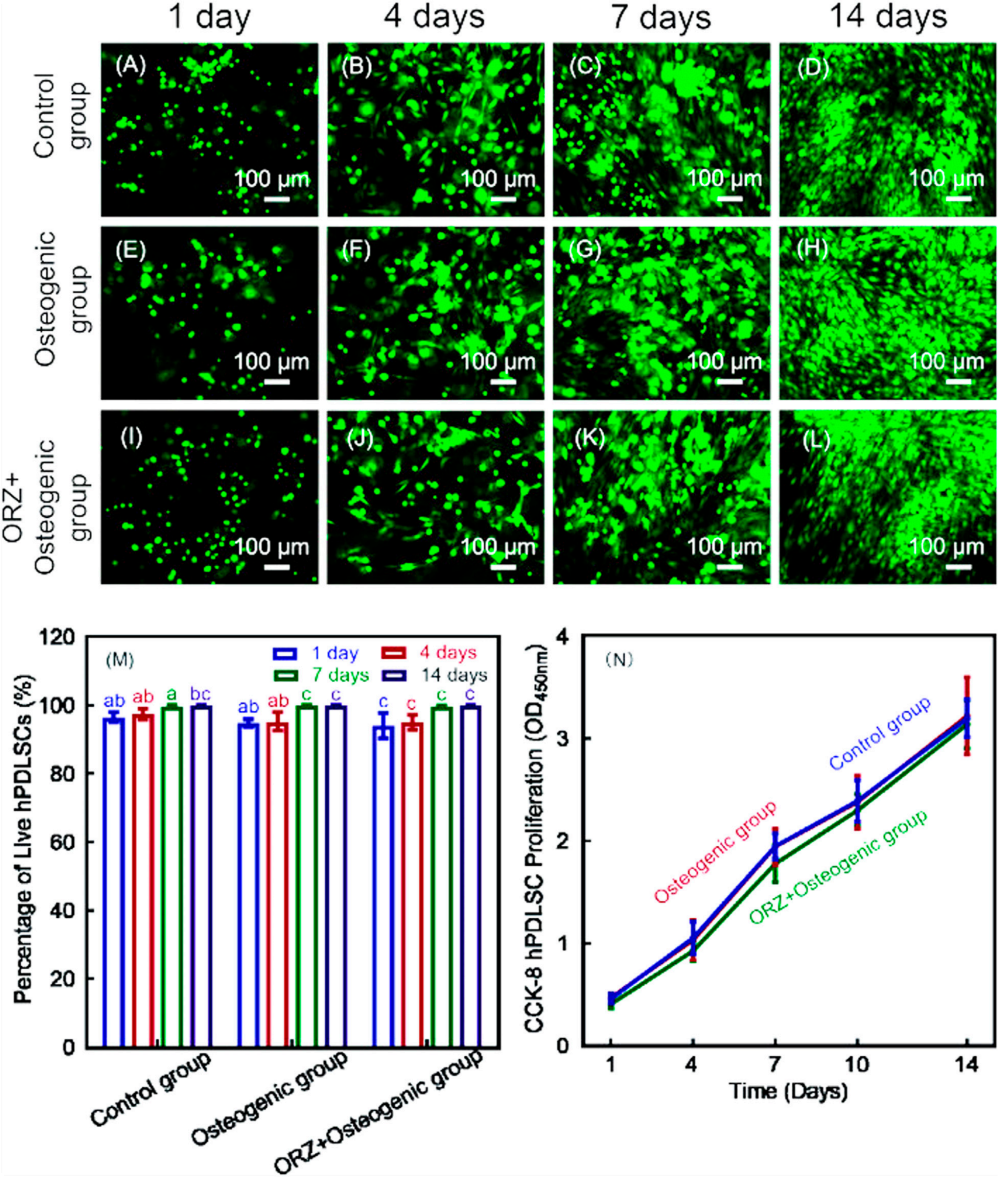
Live/dead images of PDLSCs encapsulated in alginate microbeads in CPC-chitosan scaffolds **(A–L)**. Live cells (green) were numerous and dead cells (red) were very few. Percentages of live cells **(M)** and CCK-8 from 1 to 14 days showed good cell viability and proliferation, and cell viability **(N)**. Values with dissimilar letters are significantly different from each other (*p* < 0.05).

To further increase the bone repair capability of CPC, alginate hydrogel was introduced into CPC scaffolds for cell delivery (Wang et al., 2016). The alginate hydrogel can encapsulate cells and protect them during the CPC mixing and setting reaction (Zhao et al., 2010). Moreover, after the CPC has set, the alginate hydrogel would degrade and release the seed cells and create macropores throughout the entire CPC scaffold (Zhao et al., 2010). Chen et al. (2020a) developed an injectable CPC-chitosan scaffold with PDLSCs in alginate microbeads. CPC-alginate-PDLSCs construct exhibited excellent injectability and mechanical strength (Chen et al., 2020). The released PDLSCs were able to adhere to the surfaces of CPC scaffolds and proliferate well (Chen et al., 2020) (Figure 2).

Polymeric materials include natural polymers and synthetic polymers. Natural polymers such as collagen, chitosan, gelatin, fibrin, zein and alginate have been used in tissue engineering (Zhou et al., 2012; Chen et al., 2016; E et al., 2016; Fu et al., 2016; Kammerer et al., 2017; Ou et al., 2019; Arpornmaeklong et al., 2021; Tian et al., 2021). As these natural polymers can be fabricated as hydrogels, they would mimic the chemical and physical properties of natural extracellular matrix (ECM) to enhance the osteodifferentiation of the seed cells and promote bone regeneration (Chen et al., 2016; Wang et al., 2016; Thanasrisuebwong et al., 2020; Arpornmaeklong et al., 2021; Tian et al., 2021). When seeded with PDLSCs, these natural polymers showed excellent biocompatibility, and supported the proliferation and growth of the PDLSCs (Chen et al., 2016; E et al., 2016; Fu et al., 2016; Kammerer et al., 2017; Ou et al., 2019; Arpornmaeklong et al., 2021; Tian et al., 2021). Specific applications in bone tissue regeneration require certain modifications to the polymer structure (Bharadwaz and Jayasuriya, 2020). This is quite difficult for natural polymers, and hence synthetic polymers such as PLA [poly (lactic acid)], PLGA [poly (lactide-co-glycolide)] and polycaprolactone (PCL) offer excellent applicability in bone regeneration (Alizadeh-Osgouei et al., 2019; Zhao et al., 2020; Ding et al., 2021). However, many polymeric materials suffer from the problems of weak mechanical strength (Tian et al., 2021). Moreover, some synthetic polymers, such as PLA, suffer from shortcomings including low degradation rate, low cell adhesion and inflammatory reactions due to their degradation product lactic acid (Tajbakhsh and Hajiali, 2017).

Blending of HA or nanoHA with polymers can enhance the mechanical strength and cell reaction of polymer materials (Tian et al., 2021). It was reported that sodium alginate/gelatin/nanoHA, zein/gelatin/nanoHA, collagen/nanoHA, PLA/HA displayed high potential as a tissue engineering material for bone defect reconstruction when seeded with PDLSCs (He et al., 2011; E et al., 2016; Fu et al., 2016; Ou et al., 2019; Tian et al., 2021).

## Treatments of PDLSCs with drugs and bioactive agents

In recent years, drug repurposing has become a hot pot in bone tissue engineering. Drug repurposing is concerned with the identifying of new pharmacological indications of existing drugs and their application in the treatment of diseases except for the drug’s proposed therapeutic use (Divya et al., 2021). Aspirin (non-steroidal anti-inflammatory drug) (Abd Rahman et al., 2016), metformin (hypoglycemic drug) (Zhao et al., 2019b; Jia et al., 2020), simvastatin (cholesterol-lowering drug) (Zhao and Liu, 2014; Zhao et al., 2020) and bisphosphonates (osteoporosis drug) (Zhou et al., 2011) are all Food and Drug Administration approved drugs. These drugs were all identified to induce the osteogenic differentiation of PDLSCs (Zhao and Liu, 2014; Abd Rahman et al., 2016; Zhao et al., 2019b; Di Vito et al., 2020; Jia et al., 2020; Zhao et al., 2020). Many herbals of traditional Chinese medicine (TCM) were also widely acknowledged in drug discovery and development due to their function of promoting cell proliferation and modulating bone metabolism (Liu et al., 2018). So far, multiple of active components derived from natural herbs have been discovered to have positive effects on osteogenesis, such as osthole (Gao et al., 2013; Sun et al., 2017), zanthoxylum schinifolium (Kim et al., 2015), berberine (Liu et al., 2018), naringin (Wei et al., 2017) and ginsenoside Rg-1 (Yin et al., 2015). These components of herbs were identified to enhance the proliferation or osteogenic differentiation of PDLSCs, and they have the potential to be used as a mediator or therapeutic agent in PDLSC-based bone tissue engineering (Gao et al., 2013; Kim et al., 2015; Yin et al., 2015; Sun et al., 2017; Wei et al., 2017; Liu et al., 2018). Nevertheless, functions of many drugs are complicated and need to be explored further. Furthermore, the mechanisms of many herbal-drug and their derivatives are still not well understood, which hampers their clinical application in bone regeneration.

Vitamins and growth factors are both essential biologics that skeleton requires (Myneni and Mezey, 2017; Atarbashi-Moghadam et al., 2022). Vitamins, like Vitamin C, Vitamin D and Vitamin P, have been proven to improve the osteogenic properties of PDLSCs, including promoting proliferation, increasing ECM synthesis, enhancing expression of osteogenic markers or reducing inflammation (Wei et al., 2012; Zhao et al., 2019; Qi et al., 2021). However, both excessive and insufficient usage of vitamins may be associated with compromised bone formation (Ahmadieh and Arabi, 2011). Further investigations are still needed on the proper usage methods of vitamins in PDLSCs-based bone regeneration. Growth factors have been proven to have positive effects on the osteogenesis of PDLSCs, including stromal cell-derived factor (SDF) (Liang et al., 2021), transforming growth factor (TGF) (Maeda et al., 2013; Hyun et al., 2017; Huang et al., 2021), bone morphogenic protein (BMP) (Hyun et al., 2017), vascular endothelial growth factor (VEGF) (Lee et al., 2012), fibroblast growth factor (FGF) (Lee et al., 2012; Lee et al., 2015), progranulin (PGRN) (Chen et al., 2020; Yu et al., 2021), estrogen (E et al., 2016), oxysterol (Lee et al., 2017) and erythropoietin (EPO) (Zheng et al., 2019). Further studies in this field should be dedicated to determining the appropriate dosage and timing for the addition of growth factors to different cell culture environments (Hyun et al., 2017).

**FIGURE 3:**
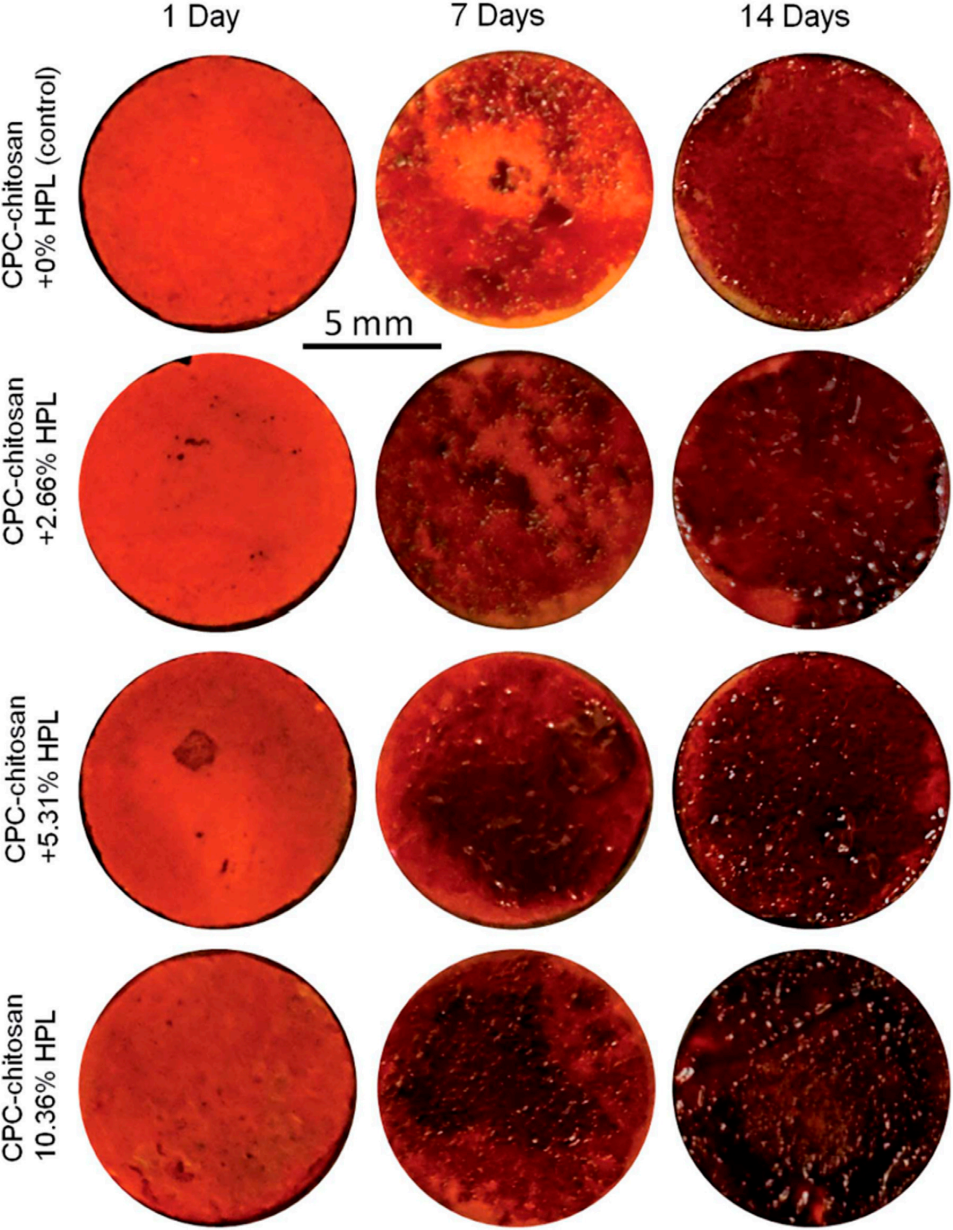
Representative alizarin red staining images of bone mineral synthesis by PDLSCs on CPC. The red staining for the HPL groups was deeper and denser that without HPL at 7 and 14 days, as the mineralization was enhanced by incorporating PL into CPC.

**FIGURE 4:**
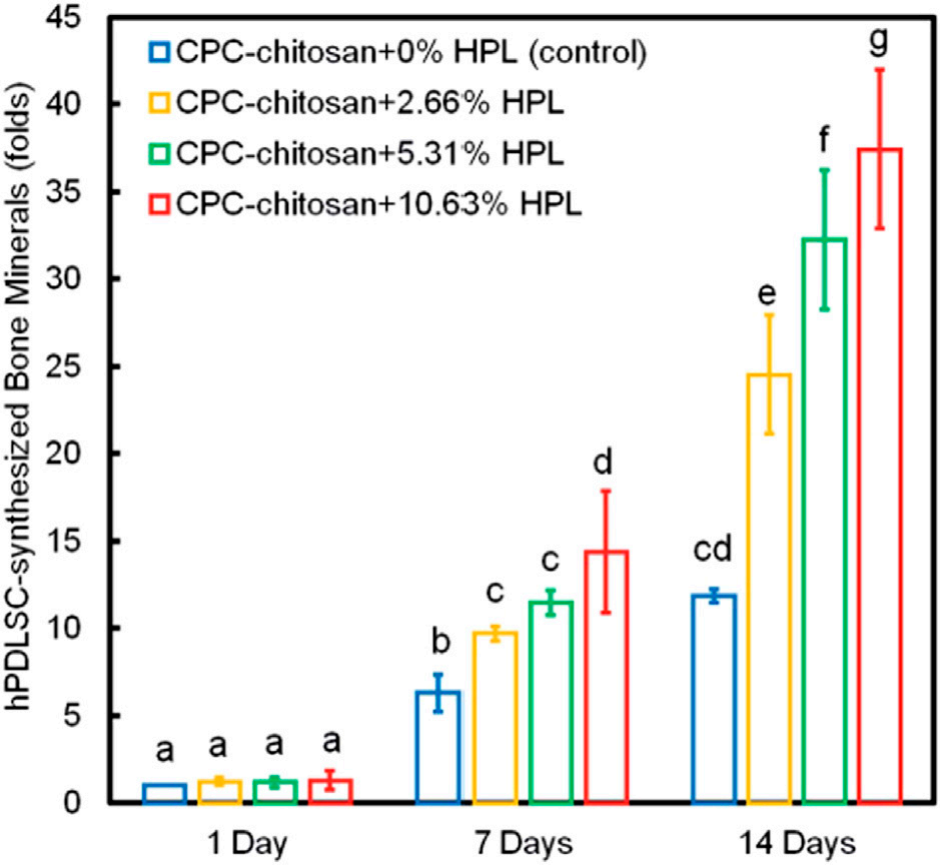
PDLSC synthesis of bone minerals. The HPL groups accumulated more minerals than that at 0% HPL. Values with dissimilar letters are significantly different from each other (*p* < 0.05).

Bone regeneration is a complicated process which involves a variety of biologics (Zhao et al., 2019c). Several animal-derived reagents were applied in bone regeneration, including platelet-rich plasma (PRP), HPL, enamel matrix derivative (EMD) and exosomes. PRP and HPL contain a cocktail of growth factors, like FGF, VEGF, TGF, platelet-derived growth factor (PDGF) and insulin-like growth factor (IGF) (Zhao et al., 2019c). Xu et al. (2017) reported that PRP at a concentration of 1% was found to improve osteogenic differentiation, cell sheet formation and ECM production of human PDLSCs. Zhao et al. (2019c) reported that CPC-chitosan scaffold was a promising vehicle for HPL delivery, and HPL exerted excellent induction on PDLSCs for bone regeneration (Figures 3, 4). Enamel matrix derivative (EMD) is an acidic extract of extracellular enamel matrix (Tanimoto et al., 2012). Several previous studies demonstrated the effect of EMD on the osteogenesis including the induction of multiple bone markers, such as bone sialoprotein (BSP), osteocalcin (OCN) and alkaline phosphatase (ALP) (Tanimoto et al., 2012). Wang et al. (2016b) revealed that EMD-enhanced PDLSC sheets secreted richer ECM, and induced higher mRNA expression of osteogenic genes than the normal PDLSC sheets. Exosomes are extracellular vesicles with diameters that ranged from 30 nm to 120 nm (Petho et al., 2018). These lipid bilayer-enclosed vesicles transfer proteins, lipids, and noncoding RNAs (Al-Sowayan et al., 2020). The findings of Wang et al. (2020) indicated that the exosomes from human exfoliated deciduous teeth promoted PDLSCs osteodifferentiation and mineralization *via* Wnt/β-catenin and BMP/Smad signaling pathways. As PRP, PL, EMD and exosomes contain a multitude of molecules, further study is needed to clarify which specific contents are responsible for the observed osteoinductive effects (Albanese et al., 2013; Miron et al., 2014; Altaie et al., 2016; Wang and Thomsen, 2021).

## Gene modulation of PDLSCs

Gene modulation is a highly promising approach to promote bone regeneration. An abundance of previous data revealed that gene therapy could be applied to successfully help repair bone defects (Shapiro et al., 2018). By gene therapy, a variety of therapeutic genes can be delivered to stimulate bone regeneration (Lu et al., 2013). Distinct from direct protein delivery (such as growth factors), genes delivery supplies a potential means to enable protein expression for a longer time and temporal modulation of the transgene expression (Lu et al., 2013). Gu et al. (2020) used the green fluorescence protein lentivirus infection system to overexpress TAZ (a key regulator of osteogenesis) in PDLSCs to promote their osteogenesis. In the study of Jung et al. (2014), the osteogenesis of PDLSCs were effectively enhanced after being transduced using replication deficient recombinant adenovirus (rAd) encoding BMP-2. These findings indicate that gene delivery represented a novel and promising strategy for promoting the PDLSCs-based bone tissue engineering.

Besides genetic transformation to express particular protein-coding genes, one of the recent promising gene therapies to be investigated for bone tissue engineering is the use of regulatory noncoding RNAs (ncRNAs). Regulatory ncRNAs include microRNAs (miRNAs), long noncoding RNAs (lncRNAs), and circular RNAs (circRNAs). These regulatory ncRNAs can regulate DNA transcription and translation (Liu et al., 2019).

The miRNAs regulate many biological processes, including cell differentiation, proliferation, angiogenesis, and apoptosis (Pizzicannella et al., 2018). Currently, several miRNAs have been identified to induce the osteogenic ability of PDLSCs, including miRNA-214 (Cao et al., 2017) and miRNA-210 (Pizzicannella et al., 2018). Cao et al. (Cao et al., 2017) revealed that miRNA-214 promoted PDLSCs osteoblastic differentiation by regulating the Wnt/β-catenin signaling. Pizzicannella et al. (Pizzicannella et al., 2018) found that miRNA-210 mediated VEGF upregulation in PDLSCs.

Previous studies supported the possibility that lncRNA and circRNA may act as competing endogenous RNAs for miRNAs to regulate their targeting gene expression in the physiological and pathophysiological process of MSCs (Gu et al., 2017; Wu et al., 2020). For instance, Jia et al. (2015) promoted osteogenic differentiation of PDLSCs *via* down regulating anti-differentiation ncRNA, a lncRNA that keeps MSCs remain an undifferentiated cell state. Wu et al. (2020) found that overexpression of lncRNA-TUG1 can accelerate the osteogenic differentiation of PDLSCs through sponging miRNA-222-3p to negatively regulate Smad2/7 signaling. Gu et al. (2017) reported that circRNA-BANP and circRNA-ITCH were predicted to interplay with miRNA-34a and miRNA-146a to modulate PDLSC osteogenesis *via* the MAPK signaling pathway. More research is needed to investigate the safety of gene therapy, especially regarding biodistribution, toxicity, and tumorigenicity (Shapiro et al., 2018).

## PDLSC cell sheets

Cell sheet technique was based on culturing cells in hyperconfluency until they form extensive cell interactions and produce ECM (Yorukoglu et al., 2017). Cell sheets have a high cell density and a uniform cell distribution and thus can closely mimic native tissue (Lu et al., 2019). PDLSC sheet has been used clinically for bone regeneration and a few clinical studies revealed that patients treated with PDLSC sheets exhibited significant bone repairing in the alveolar bone (Zhang et al., 2021). However, as cell sheets possess weak mechanical strength, rebuilding bone tissue with cell sheets alone still remains a challenge (Lu et al., 2019). Thus, many studies have investigated the bone regeneration by combining cell sheets with scaffolds, which could supply initial mechanical strength and spatial integrity (Lu et al., 2019).

Multiple types of scaffolds have shown the potential of its therapeutic use with PDLSC sheets to improve bone repair, like HA-TCP (Gao et al., 2013), collagen (Safi et al., 2019), fibrin (Wang et al., 2016) and PCL (Zhao et al., 2020). However, the main limitation of cell sheet-based tissue engineering is the possible necrosis inside the cell sheet due to the insufficient nutrient and oxygen supply and the poor exchange of cell waste (Lu et al., 2019). Therefore, recent works focus mainly on the vascularization of cell sheet engineering (Yorukoglu et al., 2017). Currently, the constructed cell sheets usually involved a large number of cells while with a relatively small proportion of ECM, which are quite different from native tissues and is a challenge requiring further investigation (Lu et al., 2019).

## Scaffold prevascularization with PDLSCs

Angiogenesis is vital for a successful therapeutic outcome in bone regeneration (Chen et al., 2018). Scaffold prevascularization is one of potential ways to increase the supply of inadequate oxygen and nutrition in implantation area (Chen et al., 2018). Endothelial cells (ECs) are main cellular component of the capillary walls, but ECs alone can only form incipient microvascular structures that resemble early capillaries (Liu et al., 2017). While vascular structures derived from co-cultured MSCs and ECs were proved to be stable (Liu et al., 2017). MSCs can secrete angiogenic growth factors (like VEGF and FGF) to induce the angiogenesis of ECs and form a pericyte-like coverage around endothelial tubes, which enables the immature vessels to remain stable (Zhao et al., 2021). Thus, co-culturing of MSCs and ECs is promising to achieve the prevascularization of scaffolds.

**FIGURE 5:**
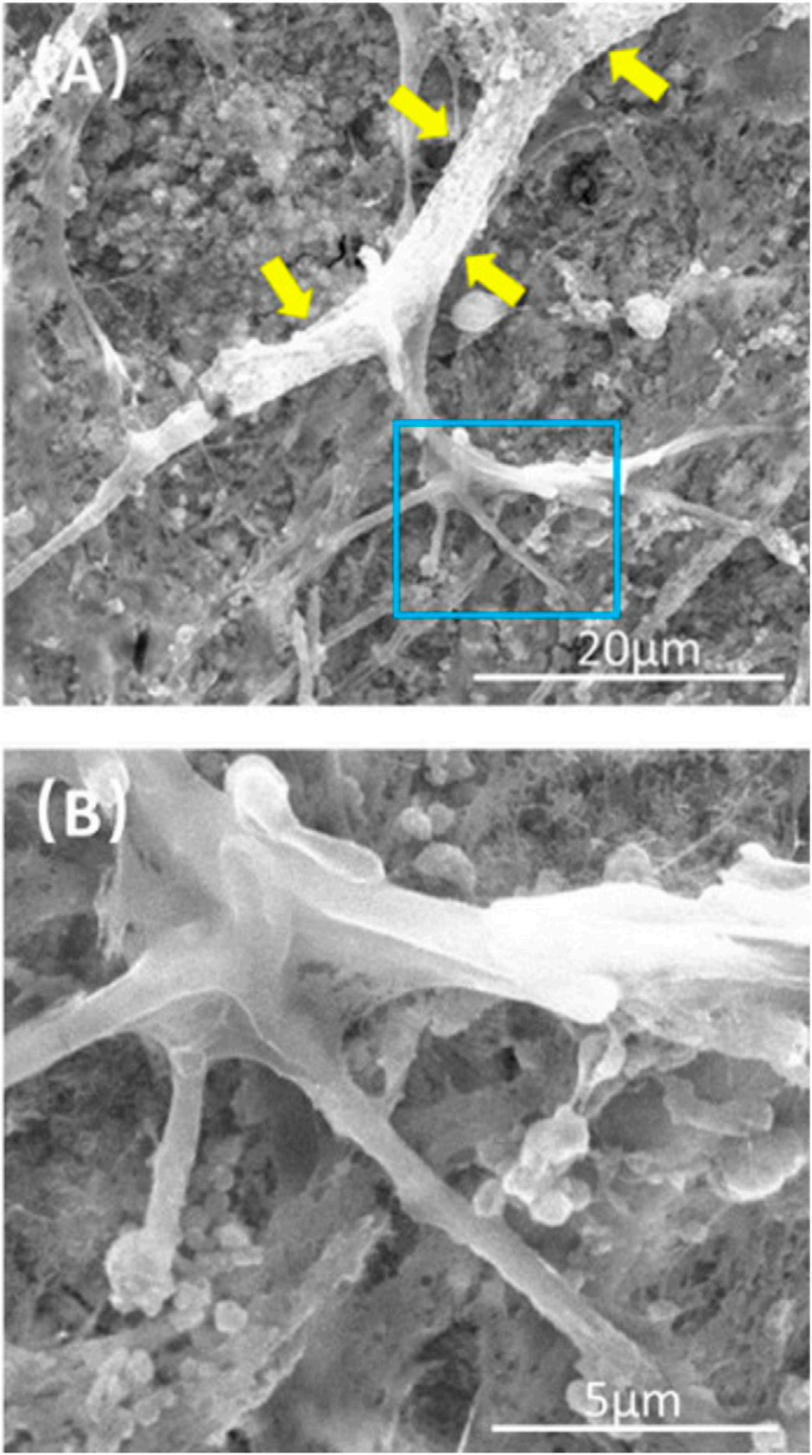
Representative SEM images of CPC scaffolds at 21 days. **(B)** Is a higher magnification image of the blue dotted frame in **(A)**. Capillary-like structures can be observed on the surface of CPC scaffolds (yellow arrows), and some branch-like stretches were found.

**FIGURE 6:**
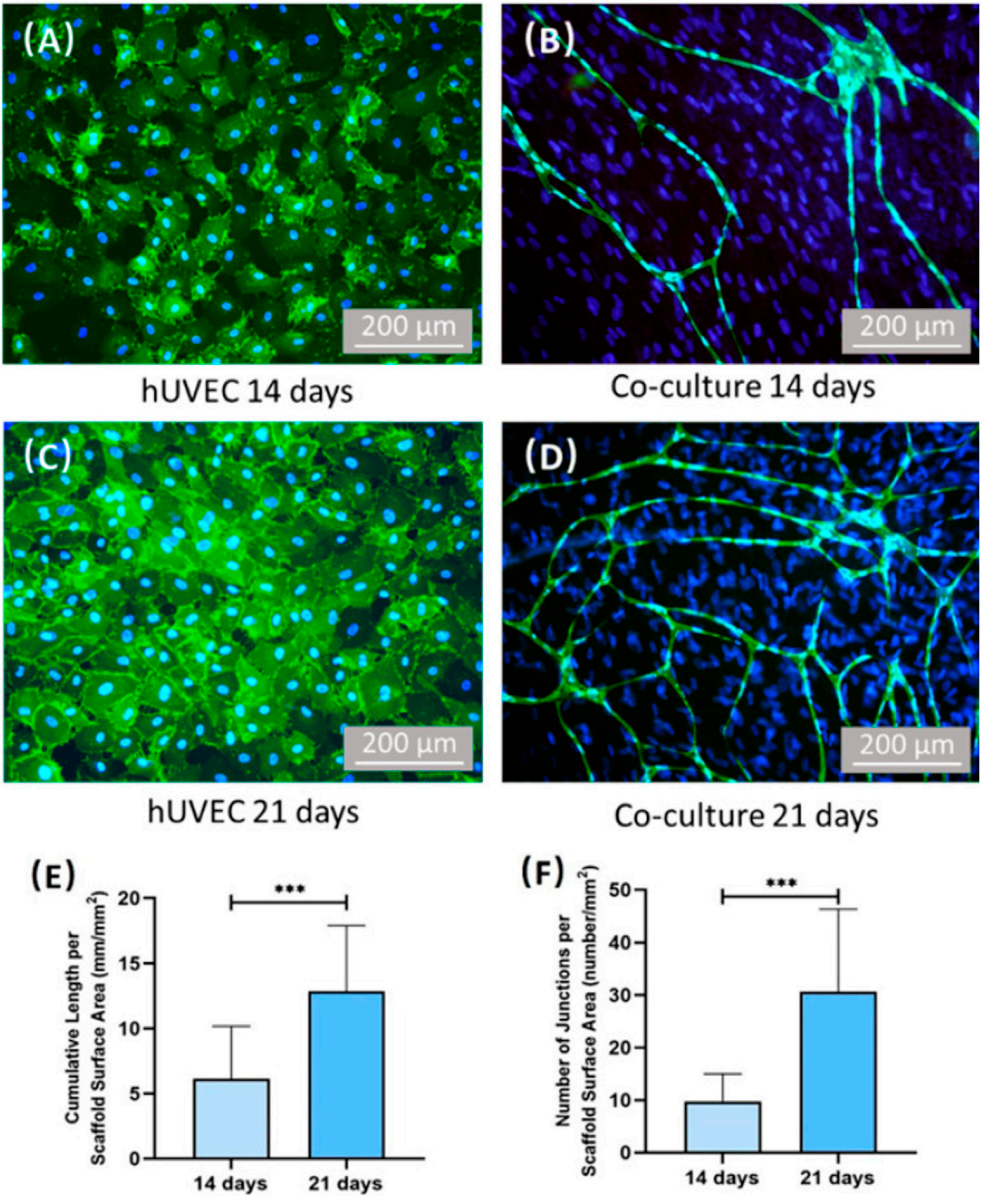
Images of CD31 immunostaining and quantification of vessel length/junctions. Human umbilical vein endothelial cells (hUVECs) were identified by immunostaining with CD31 in green on the cell membrane, and the nuclei were stained with DAPI in blue. PDLSCs were stained by nuclei counterstaining with DAPI in blue but without green stains on the cell membrane. For hUVEC group, no capillary-like structure can be found after culturing 14 days. **(A,C)** Co-culture group formed capillary-like structures after culturing 14 days. **(B,D)** Additionally, the vessel length and vessel junction number of co-culture group increased with time. **(E,F)** ***On the top of indicated statistical significance between groups (*p* < 0.001).

PDLSCs had vascular potential and were able to initiate *in vitro* angiogenesis of ECs (Yeasmin et al., 2014). Zhao et al. (2021b) co-cultured PDLSCs and ECs on CPC scaffolds and successfully formed microvascular-like structures (Figure 5). Their study indicated that the PDLSC-EC co-culture had better angiogenesis than monoculture (Zhao et al., 2021) (Figure 6). This indicated that CPC scaffolds prevacularization *via* PDLSC-EC are promising for enhancing bone tissue repairing. However, vessel network maturation and graft-host vessel anastomosis still remain the most critical challenges facing scaffold prevascularization, and further studies are needed to investigate PDLSC-based angiogenesis in animal models.

## Physical stimulation and scaffold modification for PDLSCs

Physical therapies have been popular in bone repair yield for many years due to its safety, non-invasiveness, economic benefits, easy access and controllability (Liu et al., 2022). Previous studies have shown that various kinds of physical stimuli contribute to osteogenic differentiation of PDLSCs, including mechanical stimulation, ultrasound stimuli and light stimuli (Zhang et al., 2012; Zhang et al., 2016; Li et al., 2021; Liu et al., 2022). Zhang et al. (2016) treated PDLSCs with static stress and found that exposing PDLSCs to hydraulic pressure enhanced their osteodifferentiation *via* regulating RANKL/OPG ratio by the Wnt/β-catenin pathway. Zhang et al. (2012) observed an increase in PDLSCs markers of osteogenesis after low-magnitude, high-frequency mechanical vibration stimulation. According to Li et al. (2021), low-intensity pulsed ultrasound can increase the ECM synthesis, osteogenic differentiation-related genes and proteins of PDLSCs that seeded on HA scaffolds. Yamauchi et al. (2018) revealed that 650-nm high-power red light-emitting diode increased PDLSCs proliferation, and osteogenic differentiation and mineralization by activating ERK1/2 signaling pathway. However, so far, the effect of most physical stimulation on PDLSCs is still at the preclinical stage. Further clinical trials about these physical stimulations are needed to test the optimal experimental conditions, including stimulus intensity, duration, and application frequency (Liu et al., 2022).

Surface topography on biomaterial scaffolds played an important role in regulating cell attachment, proliferation, differentiation and gene expression (Mao et al., 2015). Surface modification with micro/nano structures have been identified to be an effective method to further increase the regulating cell differentiation and cellular responses of scaffolds (Mao et al., 2015; Fu et al., 2016; Tansriratanawong et al., 2018; Daghrery et al., 2021). Daghrery et al. (2021) found that nanostructured fluorinated calcium phosphate coatings on PCL scaffolds upregulated osteogenic genes of seeded PDLSCs. Mao et al. (2015) fabricated HA bioceramics with micro-nano-hybrid surface (mnHA [the hybrid of nanorods and microrods]). Their data indicated that mnHA enhanced the cell attachment, spreading, proliferation, ALP activity and cementogenic differentiation of PDLSCs (Mao et al., 2015). Tansriratanawong et al. (2018) fabricated a novel form of calcium hydrogen phosphate with a HA-like surface (CHP-HA). PDLSCs cultivated with this novel CHP-HA show better attachment, proliferation and osteogenic differentiation than CHP alone (Tansriratanawong et al., 2018). Further research is needed to demonstrate the mechanisms of fate changes induced by nanosurface properties as well as the *in vivo* bone regeneration potential of PDLSC-based scaffolds.

## Calvarial bone regeneration *via* PDLSCs

Calvarial defect models are frequently used in bone regeneration research (Bigham-Sadegh and Oryan, 2015). Allogenic grafts, calcium phosphatase scaffolds and polymers have been combined with PDLSCs to repair calvarial defects in animals (Ge et al., 2012; Diomede et al., 2016; Yi et al., 2016; Ou et al., 2019). Diomede et al. (2016) suggested that *in vivo* implantation of PDLSCs-based porcine cortico-cancellous construct in the calvaria evidenced a precocious osteointegration and vascularization process. The presence of PDLSCs could potentiate the regenerative performance of the scaffolds (Diomede et al., 2016). Chitosan/nanoHA scaffold and Zein/gelatin/nanoHA scaffold seeded with PDLSCs showed more new bone formation than the scaffold groups without PDLSCs in a calvarial defect model (Ge et al., 2012; Ou et al., 2019). However, Yi et al. (2016)’s findings suggested that the *in vivo* bone regenerative potential of PDLSCs-based BCP could be compromised in a critical-size rat calvarial bone defect model. They indicated that the inhibitory regulation of the osteogenic effect possibly caused by soluble factors released from PDLSCs, such as chordin and PDL-associated protein-1 (Yi et al., 2016).

## Alveolar bone and periodontal regeneration *via* PDLSCs

As PDLSCs can actively differentiate into osteoblast-like cells, cementoblasts and fibroblasts, many researchers investigated the effectiveness of PDLSC-based grafts in regenerating alveolar bone and periodontal tissue (Trubiani et al., 2019). It was reported that PDLSC-seeded bovine-derived bone mineral, β-TCP, BCP, gelatin sponge and PLA/PLGA formed more bone formation than groups without scaffolds or cells after implanted into alveolar bone defect (Han et al., 2014; Su et al., 2015; Chen et al., 2016; Shi et al., 2018; Ding et al., 2020; Ding et al., 2021). Furthermore, these PDLSCs-based scaffolds facilitated the regeneration of the periodontal ligament and cementum tissue *in vivo* (Han et al., 2014; Su et al., 2015; Shi et al., 2018; Ding et al., 2020; Ding et al., 2021). In periodontal tissue regeneration, challenges remain in regenerating bone-PDL-cementum complex (Liu et al., 2019). The oriented PDL would need to be inserted into newly formed cementum-like tissue and the alveolar bone, which is especially difficult to achieve in the laboratory (Liu et al., 2019).

**FIGURE 7:**
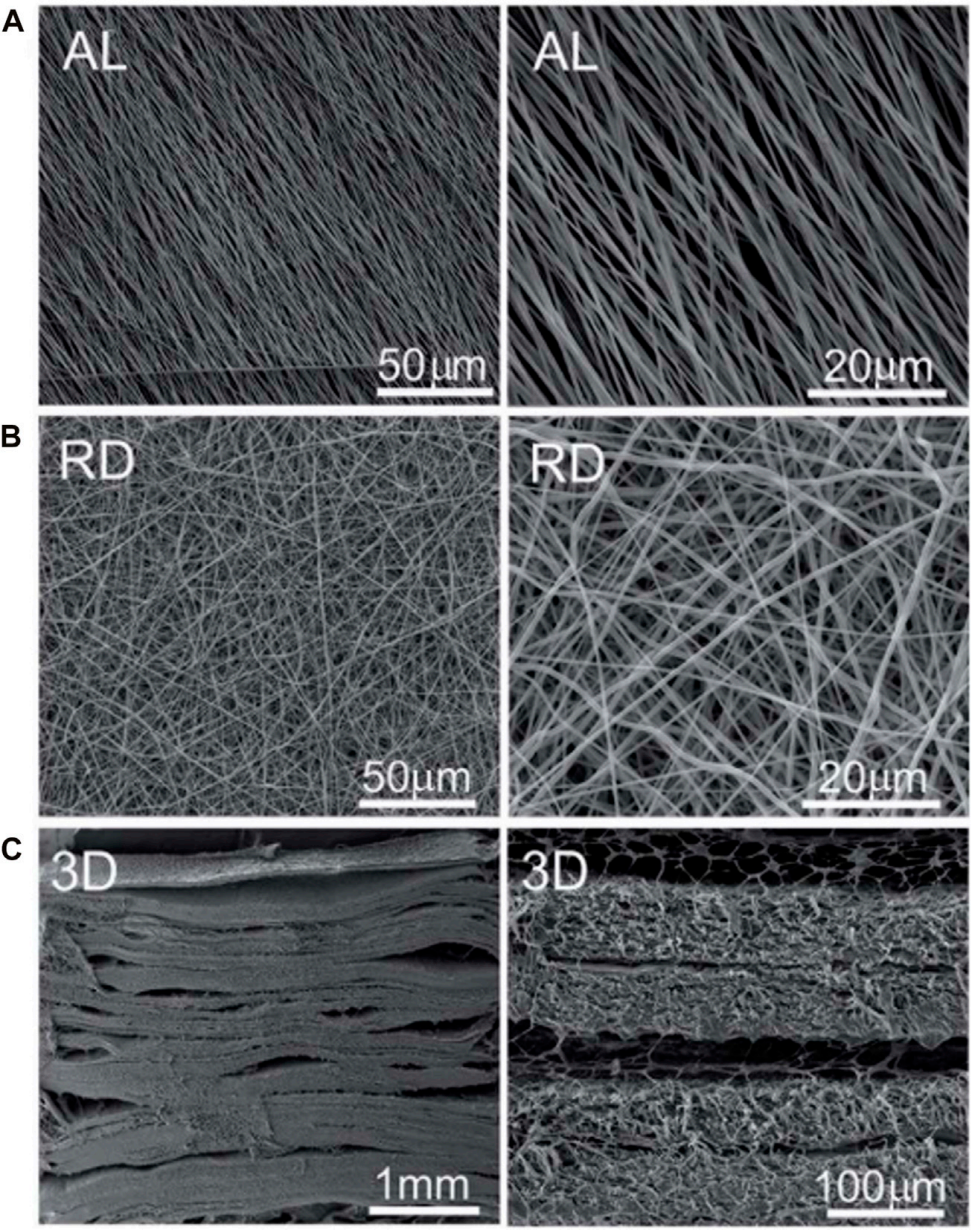
SEM photographs of 2D and 3D scaffold. **(A)** Surface topographies of aligned (AL) nanofibre. **(B)** Surface topographies of random (RD) nanofibre. **(C)** Transversal image of 3D scaffold.

**FIGURE 8:**
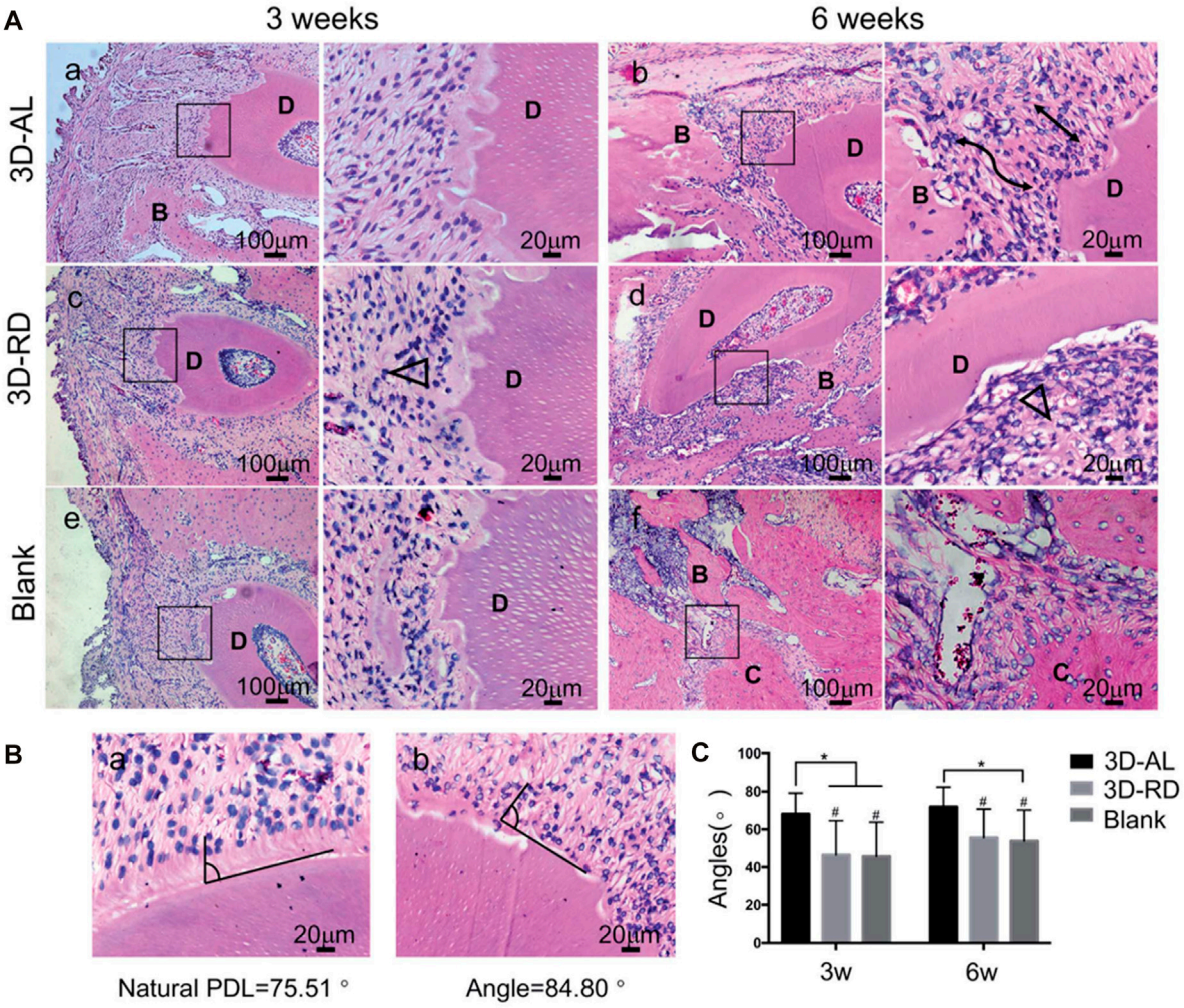
Evaluation of arrangement of regenerated ligament tissue. **(A)** Hematoxylin and eosin (H and E) staining of 3D-AL 3D-RD and blank control group at 3 and 6 weeks. The images on the right are higher magnifications of images in the black frames on the left. Black lines indicated well aligned regenerated PDL-like tissue. Black triangle represented random and irregular ligament orientation. “D” denoted “dentin,” “B” denoted “bone,” “C” denoted “cementum.” **(B)** Statistical analysis of angular distribution of regenerated PDL-like tissue in different groups. *On the top of indicated statistical significance between groups (*p* < 0.05). # On the top suggested statistical difference (*p* < 0.01) in comparison to natural PDL.

Tomography properties of scaffolds effect on periodontium regeneration (Lee et al., 2014). Gao et al. (2015) found that titania nanotubes (NTs) layered on titanium/HA scaffolds enhanced the periodontal regeneration of PDLSCs, leading to the formation of collagen fiber bundles. It indicates that the effect of nanotopographical cues can influence the functions of PDLSCs-based periodontal regeneration (Gao et al., 2015). Topographical cell guidance facilitates the geometric design of composite materials (Yang et al., 2018). It has been utilized as a tissue bionic technique in periodontal regeneration (Yang et al., 2018). Yang et al. (2018) manufactured multilayered scaffolds by cementing aligned PCL nanofibers together with gelatin (Figure 7). The scaffold mimicked the natural structure of periodontal ligaments (Yang et al., 2018). This scaffold could provide good attachment for PDLSCs (Yang et al., 2018). Yang et al. (2018) evaluated the angular distribution of regenerated PDL-like tissue by the arrangement of nuclear and cell shapes against the root surfaces, and compared it with that of physiological periodontal tissue. The *in vivo* results demonstrated that construct seeded with PDLSCs could oriented neogenesis of periodontium and facilitate collagen formation and maturation at periodontal fenestration defects (Yang et al., 2018) (Figure 8).

The favorable regenerative microenvironment was essential for inducing PDLSCs migration, differentiation, proliferation in periodontal tissue regeneration (Zhu et al., 2017). An optimal combination of suitable growth factors/cytokines and the sequential release at the right time could favor periodontal tissue regeneration (Ding et al., 2020). Cho et al. (Cho et al., 2016) used 3D-printing to fabricate PCL/PLGA scaffolds encapsulating bone morphogenetic protein-7 (BMP-7). Then the scaffolds were cultured with PDLSCs and placed on the exposed dentin (Cho et al., 2016). The results indicated that scaffolds can constantly release BMP-7 and show promise for the ability for guiding PDL and alveolar bone regeneration (Cho et al., 2016). Ding et al. developed a fibrous framework with a PLA/PLGA core/shell structure with rapid release of basic fibroblast growth factor (bFGF) and the relatively slower and sustained release of BMP-2 (Ding et al., 2020). PLA was used as the core material because of its longer degradation time, while PLGA was designed as the shell material due to its faster degradation rate (Ding et al., 2020). This sequential delivery system could stimulate the recruitment of PDLSCs, which could not only achieve bone regeneration but also restore the cementum and PDL to their native structures (Ding et al., 2020).

The cross-talk between PDLSCs and other types of cells likely affect the outcome of PDLSC-based periodontal regeneration by regulating cell growth and differentiation (Yu et al., 2017). These interactions may exist through secreted biologics or/and direct cell-to-cell contact (Yu et al., 2017). Osteoblast progenitors (Yu et al., 2017), jawbone-derived MSCs (Zhu et al., 2017) and apical tooth germ cells (Yang et al., 2009) were all found to promote the periodontium regeneration of PDLSCs. PDLSCs sheets offers advantages by secreting ECM, which provides a wide range of biochemical and mechanical cues to the cells and acts as a reservoir for many signal molecules that are critical in periodontal regeneration (Guo et al., 2014).

3D PDLSC pellets were formed by tightly condensed PDLSC sheets (Guo et al., 2014). The collagen fibers in 3D PDLSC pellets increased logistically and oriented more regularly, thus making it less resistant to stretch (Guo et al., 2014). 3D multilayered PDLSC pellet was capable to reconstruct the physiological architecture of the cementum/periodontal ligament complex (Guo et al., 2014).

## Ectopic bone regeneration *via* PDLSCs

Ectopic bone formation refers to the ossification of tissues outside their usual origins (Scott et al., 2012). Ectopic bone formation has unique advantages over orthotopic environments, including a relative lack of bone cytokine stimulation and cell-to-cell interaction with host bone-forming cells (Scott et al., 2012). Subcutaneous implantation is the most commonly used ectopic bone formation method in bone tissue engineering (Scott et al., 2012). Many types of scaffolds have been seeded with PDLSCs and transplanted subcutaneously, including polymers, calcium phosphate scaffolds and xenografts (He et al., 2011; Moshaverinia et al., 2013; Yu et al., 2014; Chen et al., 2016; E et al., 2016; Wang et al., 2016). PDLSCs were suitable seed cells for ectopic bone regeneration in the context of gelatin methacrylate/nanoHA, collagen/PLA/nanoHA, PLA/nanoHA, TCP/HA, alginate and anorganic deproteinized bovine bone minerals, and constructs promoted better bone regeneration than scaffold alone (He et al., 2011; Moshaverinia et al., 2013; Yu et al., 2014; Chen et al., 2016; E et al., 2016; Wang et al., 2016).

There is preclinical evidence that the use of PDLSC-based scaffolds improves osteogenesis when treating bone defects. However, some studies also demonstrated that the complexity of osteoinductive function of PDLSCs depend on different *in vivo* tissue environment (Yi et al., 2016). For example, Yi et al. (2016) found that PDLSCs promoted BMP-2-induced osteogenesis in subcutaneous transplantation but negatively regulated bone formation in a critical size bone defect. Thus, more extensive investigations in a variety of pathophysiological conditions are required (Yi et al., 2016). In addition, there is limited clinical evidence that these PDLSC-based scaffolds can be used safely and effectively to repair bone defects in clinical use. Further investigation is needed to acknowledge the *in vivo* regenerative properties of PDLSCs and to make their clinical use for bone tissue engineering.

## Conclusions and perspectives

In this paper, the summarized literature, which involves scaffolds for PDLSC-based bone tissue engineering, has been reviewed. The strategies and agents used to induce PDLSC osteogenesis have also been elaborated.

The purpose of using scaffold is to mimic the structure and function of ECM, which can provide a 3D environment to enhance the attachment, population, and differentiation of cells and to supply proper biological and physical condition for bone repair (Porter et al., 2009; Qu et al., 2019). Scaffolds for delivering PDLSCs were shown in Table 1. Xenogenic bone grafts and calcium phosphate materials are widely used as bioactive biomaterials due to their similarity to the inorganic component of natural bone, excellent biological reactions to cells, and well osteoconductivity (Samavedi et al., 2013). However, much work remains to be done in further enhancing their degradability and osteoinductivity (Samavedi et al., 2013). Different from calcium phosphate materials, natural polymers are extensively used in bone regeneration due to their capability to mimic ECM (Bharadwaz and Jayasuriya, 2020). They have specific degradation rates and superior biological properties (Bharadwaz and Jayasuriya, 2020). Nevertheless, most natural polymers have weak mechanical strength (Tian et al., 2021). In addition, it is quite difficult to do certain modifications with natural polymers for specific applications in bone tissue regeneration (Bharadwaz and Jayasuriya, 2020). While synthetic polymers are much easier to be modified (Bharadwaz and Jayasuriya, 2020). But many synthetic polymers suffer from the problems including low cell adhesion and inflammatory reactions (Tajbakhsh and Hajiali, 2017). Fabricating an ideal scaffold that simultaneously possess strong mechanical characteristics, interconnected porosis, well osseointegration, vascularization, controlled biodegradability and remodeling ability is still a challenge (Zhao et al., 2021). Composite biomaterials that designed to combine two or more materials might be a promising approach to increase the performance of these scaffolds (Samavedi et al., 2013).

**TABLE 1:** Scaffolds for delivering PDLSCs.

Type	Name	Characteristic	Outcome	Reference
Xenogenic bone grafts	Inorganic deproteinized bovine bone minerals (e.g. Bio-Oss)	Architectural and compositional similarities to human bone, osteoconductive, well biocompatibility.	Successfully reconstruct critical-size defects	Yu et al. (2014)
	Collagenated porcine cortico- cancellous bone scaffold (e.g. Osteobiol Dual Block)	Preserving cancellous and cortical bone structure, osteoconductive, well biocompatibility, rigid consistency.	Cells showed the highly efficient cell proliferation osteogenic differentiation, vascular differentiation and functional response.	Diomede et al. (2016), Mazzoni et al. (2017)
Calcium phosphate materials	Hydroxyapatite	Similar chemical characteristics with natural bone, excellent bioactivity and osteoconductivity.	Cell attachment, spreading, proliferation, osteogenic differentiation and cementogenic differentiation were promoted.	He et al. (2011), Mao et al. (2015)
	Tricalcium phosphate	Similar chemical constitute to natural bone, excellent osteoconductivity, porousity and fast biodegradability.	Constructs were capable to repair alveolar bone defects.	Su et al. (2015)
	Biphasic calcium phosphate	Consisted of stable hydroxyapatite phase and resorbable tricalcium phosphate phase, high biocompatibility, efficient osteoconductivity.	Cells exhibited significantly great viability. Constructs significantly promoted effective alveolar bone regeneration.	Shi et al. (2018)
	Calcium phosphate cement	Excellent biocompatibility, osteoconductivity, osteoinductivity, bioactivity and injectability.	Cells attachment, proliferation and osteodifferentiation were surppoted.	Zhao et al. (2019b), Chen et al. (2020a), Zhao et al. (2021a)
Polymeric materials	Natural polymer: collagen, chitosan, gelatin, fibrin, zein and alginate	Excellent biocompatibility, similar chemical and physical properties of natural extracellular matrix, weak mechanical strength.	The proliferation and growth of cells were surpported.	Chen et al. (2016b), E et al. (2016), Fu et al. (2016), Kammerer et al. (2017), Ou et al. (2019), Arpornmaeklong et al. (2021), Tian et al. (2021)
	Synthetic polymer: PLA [poly(lactic acid)], PLGA [poly (lactide-co-glycolide)] and polycaprolactone	Modificability, low cell adhesion and inflammatory reactions.	After blending with hydroxyapatite, constructs displayed high potential of bone regeneration.	He et al. (2011), E et al. (2016), Fu et al. (2016), Ou et al. (2019), Tian et al. (2021)

To further increase the osteogenesis of PDLSC-based bone repair, multiple strategies and agents were applied. In bone tissue engineering, three factors are essential: ideal microenvironment, appropriate scaffolds, and viable cell populations (Zha et al., 2022). Effects of drugs and bioactive agents on PDLSCs were shown in Table 2. Strategies of osteoinductive strategies on PDLSCs were shown in Table 3.

**TABLE 2:** Effects of drugs and bioactive agents on PDLSCs.

Type	Name	Effect	Reference
Drugs	Aspirin	Enhanced the proliferation, osteogenic differentiation and mineralization.	Abd Rahman et al. (2016)
	Metformin	Promoted the osteodifferentiation and mineral synthesis.	Zhao et al. (2019b), Jia et al. (2020)
	Simvastatin	Enhanced osteodifferentiation and prevented oxidative stress-induced damages.	Zhao and Liu (2014), Zhao et al. (2020)
	Bisphosphonates	Promoted the proliferation and osteogenic differentiation.	Zhou et al. (2011)
	Natural herb derivatives: Osthole, zanthoxylum schinifolium, berberine, naringin and ginsenoside Rg-1	Enhanced the proliferation or osteogenic differentiation.	Gao et al. (2013), Sun et al. (2017), Kim et al. (2015), Liu et al. (2018), Wei et al. (2017), Yin et al. (2015)
Vitamins	Vitamin C	Increased cell matrix production and ostedifferentiation.	Wei et al. (2012)
	Vitamin D	Promoted the osteogenesis and vitamin D receptor.	Qi et al. (2021)
	Vitamin P	Increased the formation and osteogenesis of cell sheet.	Zhao et al. (2019a)
Growth factors	Stromal cell-derived factor	Enhanced proliferation and migration.	Liang et al. (2021)
	Transforming growth factor	Promoted the osteogenic differentiation and ligamentogenesis.	Maeda et al. (2013), Hyun et al. (2017), Huang et al. (2021)
	Bone morphogenic protein	Stimulated osteogenic differentiation and cementogenic differentiation.	Hyun et al. (2017)
	Vascular endothelial growth factor	Promoted osteogenic differentiation and mineralization.	Lee et al. (2012)
	Fibroblast growth factor	Increased cell population and osteogenic potential.	Lee et al. (2012), Lee et al. (2015)
	Progranulin	Enhanced osteogenic differentiation. Antagonized osteogenic inhibition signaling molecules.	Chen et al. (2020b), Yu et al. (2021)
	Estrogen	Enhanced the osteodifferentiation, mineralization and in vivo bone regeneration potential.	E et al. (2016)
	Oxysterol	Increased the osteogenic activity.	Lee et al. (2017)
	Erythropoietin	Increased osteodifferentiation and mineralization.	Zheng et al. (2019)
Animal-derived reagents	Platelet-rich plasma/ platelet lysate	Enhanced the osteogenic differentiation and extracellular matrix production.	Xu et al. (2017), Zhao et al. (2019c)
	Enamel matrix derivative	Enhanced cell matrix production and ostedifferentiation.	Wang et al. (2016b)
	Exosome	Promoted osteodifferentiation and mineralization.	Wang et al. (2020)

**TABLE 3:** Effects of osteoinductive strategies on PDLSCs.

Method	Effect		Reference
Protein-coding genes modulation:	Overexpression of TAZ promoted osteogenesis		Gu et al. (2020)
	Encoding of BMP-2 enhanced osteogenesis		Jung et al. (2014)
Noncoding RNAs modulation:	miRNA	miRNA-214 promoted osteodifferentiation via regulating Wnt/β-catenin signaling.	Cao et al. (2017)
		miRNA-210 promoted osteogenesis via mediating VEGF upregulation.	Pizzicannella et al. (2018)
	lncRNA	lncRNA-TUG1 promoted osteodifferentiation through sponging miRNA-222-3p to negatively regulate Smad2/7 signaling.	Wu et al. (2020)
	circRNA	circRNA BANP and circRNA ITCH Interact with miRNA34a and miRNA146a to regulate osteogenic differentiation via the MAPK pathway.	Gu et al. (2017)
Cell sheets	Closely mimic native tissue and improve bone tissue regeneration.		Gao et al. (2013), Safi et al. (2019), Wang et al. (2016c), Zhao et al. (2020)
Scaffold prevascularization	Increase the supply of inadequate oxygen and nutrition in implantation area.		Zhao et al. (2021a)
Physical stimulation	Mechanical stimulation	Static stress enhanced osteodifferentiation via regulating RANKL/OPG ratio by the Wnt/β-catenin pathway.	Zhang et al. (2016)
		Low-magnitude, high frequency mechanical vibration promoted osteogenic differentiation.	Zhang et al. (2012)
	Ultrasound stimuli	low-intensity pulsed ultrasound increased the osteogenic differentiation and mineralization.	Li et al. (2021)
	Light stimuli	High-power red light-emitting diode increased proliferation, and osteogenic differentiation and mineralization by activating ERK1/2 signaling pathway.	Yamauchi et al. (2018)
Scaffold modification	Nanostructured fluorinated calcium phosphate coatings increased osteogenesis.		Daghrery et al. (2021)
	Nanostructured hydroxyapatite surface enhanced the cell attachment, spreading, proliferation, osteogenic differentiation and cementogenic differentiation.		Tansriratanawong et al. (2018), Mao et al. (2015)

Osteoinductive drugs/bioactive molecules/reagent were applied in the living microenvironment of PDLSCs to further enhance osteogenesis effect. These additives were immediately mixed with medium or loaded by scaffolds. Further studies in this field need to set insight on the dosage, addition time, mechanisms and possible side effects. In addition, delivery systems with an ability to locally control over spatial distribution and sustained release of these drugs or biological agents also need to be investigated (Porter et al., 2009; Oliveira et al., 2021). Physician stimulation is a low cost and noninvasive therapy that can change cell microenvironment (Liu et al., 2022). However, due to the complexity of the interaction between physical agents and biological systems, a lot work is still needed to find out the specific mechanisms (Massari et al., 2019). Furthermore, great care has to be given to the design of dedicated experimental set-ups, in which the stimulation contributions can be controlled (Padilla et al., 2014).

As interface plays a significant role in osseointegration of implanted scaffolds, surface modification can effectively enhance the osteogenesis (Walmsley et al., 2015). Nanoparticle modifications of scaffolds enhance their capacity to mimic complex properties of the natural bone and provide a more favorable milieu for cellular adherent, migration, and bone regeneration (Walmsley et al., 2015). While further in-depth structural and functional studies are required to understand the underlying mechanisms for cell responses to different surface topographies (Gui et al., 2018).

Several strategies focused on the tissue formation capability of PDLSCs, including cell sheets and scaffold vascularization. As the cellular attachment proteins and extracellular matrix were kept, PDLSC sheet yielded greater bone regeneration than single PDLSCs (Li et al., 2019). However, so far, a single cell sheet still cannot sufficiently regenerate a large-scale bone defect (Li et al., 2019). In addition, possible necrosis and insufficient ECM are still the main problems of PDLSC sheet (Yorukoglu et al., 2017; Lu et al., 2019). Scaffold prevascularization can enhance bone regeneration *via* increasing oxygen and nutrition supply and helping remove waste (Zhao et al., 2021). PDLSCs showed great vascular potential when they were co-cultured with ECs (Zhao et al., 2021). However, further investigation about vessel network maturation and graft-host vessel anastomosis are still needed. Gene therapy is a breaking new technology with the aim of regenerating tissues by acting as a delivery system for therapeutic genes (Fliefel et al., 2017). But functional investigation is necessary to find out both safety and efficacy prior to its future clinical application.

PDLSC-based constructs showed potential effect on the *in vivo* tissue regeneration of calvarial bone, alveolar bone and ectopic bone. Moreover, PDLSC-based constructs also demonstrated excellent capacity of periodontal tissue repair. This indicated that PDLSC-based construct is promising as a tissue engineering scaffold for bone regeneration, especially the repair of craniofacial bone. However, bone is a dynamic organ, which interacts with the immune system and vascular system, future research needs to focus not only on osteogenic effect, but also on immune reaction and vascular (Madsen et al., 2020). Additionally, future *in vivo* study design would center on the performance of PDLSC-based constructs in various pathophysiological environments in order to achieve optimal effects.

PDLSCs are a relatively new, readily accessible, and highly promising MSC source for bone and dental tissue engineering. PDLSCs showed higher growth potential than borrow mesenchymal stem cells (BMSCs). For example, BMSCs stopped proliferation at approximately 50 population doublings, while PDLSCs maintained the proliferative capacity even beyond 100 population doublings (Tomokiyo et al., 2019). In delivering PDLSCs, scaffolds including calcium phosphates and polymers exhibited excellent osteobiological properties for bone grafts. PDLSC-based bioactive constructs showed great potential in regenerating calvarial bone, alveolar bone, ectopic bone and the periodontal complex. Several novel approaches for PDLSCs-based bone regeneration showed excellent potential, including the co-delivery of drugs and biologics, and the employment of gene therapy, physical stimulation, cell sheets, scaffold surface modification and scaffold prevascularization. However, more standardized preclinical and clinical studies are still needed to understand their *in vitro* performance, *in vivo* effects, clinical usage and safety. Nonetheless, the novel PDLSCs-based bone grafts are highly promising for the regeneration of bone, dental and periodontal tissues.
